# Down-Regulation of MHC Class I Expression in Human Keratinocytes Using Viral Vectors Containing *US11* Gene of Human Cytomegalovirus and Cultivation on Bovine Collagen-Elastin Matrix (Matriderm^®^): Potential Approach for an Immune-Privileged Skin Substitute

**DOI:** 10.3390/ijms20092056

**Published:** 2019-04-26

**Authors:** Frederik Schlottmann, Sarah Strauss, Kevin Hake, Peter M. Vogt, Vesna Bucan

**Affiliations:** Department of Plastic, Aesthetic, Hand and Reconstructive Surgery, Hannover Medical School, Carl-Neuberg-Strasse 1, 30625 Hannover, Germany; strauss.sarah@mh-hannover.de (S.S.); hake.kevin@mh-hannover.de (K.H.); vogt.peter@mh-hannover.de (P.M.V.); bucan.vesna@mh-hannover.de (V.B.)

**Keywords:** MHC I receptor, US11 gene, primary keratinocytes, skin graft, Matriderm^®^

## Abstract

Skin transplantation, especially in burn patients, is still challenging because surgeons are faced with limited disposability of autologous donor side material. The in vitro culture of keratinocytes has become an important reconstructive option. However, only non-immunogenic allogenic keratinocytes offer the opportunity to develop a skin graft that can overcome rejection. The purpose of the study was to develop targeted gene modification of keratinocytes in order to reduce immunogenicity for the use as allogenic transplantable skin graft by decreasing the expression of MHC class I. To reduce MHC class I expression, viral vectors containing the *US11* gene of human cytomegalovirus were generated and tested on their functionality using Western blotting, indirect immunofluorescence staining, and flow cytometry. Transfected keratinocytes were seeded on commercially available bovine collagen-elastin matrices and further cultured for histological and cell survival assays. Results showed transient down-regulation of MHC class I after 24 h post-transfection, with recovery of MHC class I expression after 48 h. Histological assessments showed long-term cell survival as well as histological patterns comparable to epidermal layers of healthy human skin. The data postulates the potential application of *US11* transfected keratinocytes as an approach towards an immune-privileged skin substitute. Nevertheless, further studies and data are needed.

## 1. Introduction

The skin, one of the largest organs of the human body, fulfills a variety of functions. It plays a significant role in thermoregulation and serves as a mechanical and chemical barrier to the external environment to prevent fluid loss and microbial contamination [1]. Plastic-reconstructive surgeons are faced with various problems when treating patients who suffer from severe skin defects with a high percentage of skin surface loss. For instance, skin surface loss can co-occur due to burn injuries or chronic wounds [2]. The defect size, fluid and thermal loss as well as local and systemic inflammations due to infections can lead to life-threating conditions. Advances in burn care in the last few years have reduced morbidity and mortality rates [3]. Yet, reconstructive surgical procedures still represent a supporting pillar to fully restore the cutaneous functionality. Standardized surgical procedures focus on autologous skin transplantations such as split-thickness and full-thickness skin grafting [2,4]. Both techniques are well-established but at the same time go hand in hand with donor side morbidity and limited disposability [2,5]. Furthermore, autologous keratinocytes are commercially available as sheets (EpiCel™) [6] and sprayed cell suspension (ReCell™) [7], but share the above-mentioned disadvantages. However, especially when faced with major skin defects, allogenic or xenogenic skin grafts are used as temporary wound coverage [2,8,9]. Allogenic or xenogenic grafts share the disadvantages of immune incompatibility, secondary infection, graft rejection, and tendency of scarring [9]. Research on skin reconstruction is an important field in experimental surgery and focuses on tissue engineering of skin substitutes, transplantation of keratinocytes [10], as well as alternative techniques to autologous skin grafting [11]. Despite various approaches to optimize skin transplantation or create novel skin substitutes with high biological functionality, there is still a need for an ideal immune compatible full-thickness skin substitute that tackles all of the above-mentioned challenges.

Beside the techniques for cutaneous reconstruction mentioned above, a plethora of different commercially accessible synthetic and biological materials for temporary or permanent cutaneous coverage is available. Each of them protects open wounds, promotes regenerative processes, suppresses scarring, and has its position in clinical routine [9,12,13]. Matriderm^®^, the biological matrix used in this study, is a permanent dermal skin substitute for full-thickness skin defects. It is commercially available in various thicknesses and consists of an avital and cell-free three-dimensional bovine collagen-elastin matrix. It is clinically well-established and reveals suitable vascularization in vivo [14]. In vitro, the matrix is suitable as scaffold for cell seeding and; therefore, tissue engineering applications [15]. Various cell types, such as pancreatic stem cells [16], preadipocytes [17], fibroblasts, and keratinocytes [15,18], adhere and proliferate on Matriderm^®^. After implantation in rodents, components are remodeled to the recipients’ matrices over the time [19,20].

Another promising attempt to enhance skin reconstruction are genetically modified keratinocytes for allogenic transplantations that can overcome graft rejection and immune incompatibility. Morgan et al. first described the successful in vitro gene modification of keratinocytes by applying the method of retroviral gene transfer. They transferred a recombinant human growth hormone gene into cultured human keratinocytes, transplanted them into athymic mice and observed epithelial regeneration [21]. Another rodent study showed that mice lacking major histocompatibility complex (MHC) class I and class II molecules rejected allogenic skin grafts with little delay. Furthermore, grafts from MHC-deficient animals were rapidly rejected by normal allogenic recipients [22]. In recent years, other gene modifying techniques have been investigated, such as the use of plasmids for the transfection of keratinocytes [23]. Additionally, some studies focused on retroviral transfection of keratinocytes with human growth hormone and its in vivo application in a pig model. Over a period of four weeks, a reconstruction of the epidermis was observed and human growth hormone could be detected in wound fluids [24,25]. Over the years, especially gene therapy for the skin has been investigated intensively but has shown limited success [26]. Even further studies showed reduced graft rejection of allogenic skin transplants in *transporter-associated-with-antigen-processing* (TAP)- and *β_2_-microglobulin* (β_2_M)-deficient mice [27,28,29]. Models focusing on β_2_M- and TAP-deficient mice pointed out that the MHC class I surface expression is crucial for rejecting transplanted allogenic tissues. Whereas β_2_M-deficient mice showed indefinite survival of transplanted allogenic pancreatic islets [30], allogenic heart and liver transplantation models only showed decreased allograft rejections [31]. Due to the complex genomic diversity of MHC class I genes, the entire genomic knockout of MHC class I is not feasible. As another approach for allogenic skin transplantation with reduced rejection rates, intrabody-mediated knockout of MHC class I was investigated in monkey cell lines and primary human keratinocytes. It was observed that endoplasmic reticulum directed anti-human MHC class I single chain intrabodies could effectively block MHC class I cell surface expression in both cell lines with different human leukocyte antigen (HLA)-A, -B, and -C haplotypes [32]. This work group also investigated adenovirus-mediated transfection of human primary keratinocytes and observed noteworthy reduction of MHC class I surface expression. Based on current knowledge, there is a need for a favorable strategy for immunomodulation of keratinocytes to reduce graft rejection in terms of skin allo-transplantation.

Through evolutionary processes, many viruses have developed suitable strategies to interfere with immune recognition [33,34]. The human cytomegalovirus (HCMV), member of the beta-subfamily of herpes viruses, has developed defense mechanisms to interfere with the host cellular immune response by reducing the surface expression levels of MHC class I [35,36,37]. Therefore, HCMV affects the antigen presentation on the cell surface to CD8+ cytotoxic lymphocytes [38,39,40]. As Ahn et al. [41] showed, HCMV uses different strategies to escape T-cell recognition during the viral infectious cycle. It was first assumed and later on proved that presumably all MHC class I down-regulating genes are located within the unique short glycoprotein (US) region of *US2*, *US3*, *US6*, and *US11* as the deletion of the whole region from the viral genome has been shown to prevent down-regulation of MHC class I molecules [36,42]. Each of these proteins is expressed at specific points of the viral cycle and has individual targets within the MHC class I antigen presentation pathway. For instance in HCMV infected cells, *US2* and *US11* redirect newly synthesized MHC class I heavy chains from the endoplasmic reticulum to the cytosol where they are degraded by the proteasomes [43,44,45]. *US11* transfected cells reveal a significant reduction of MHC class I surface expression and at the same time do not affect the TAP-dependent transport of peptides [46]. However, *US2* seems to be slightly more suppressive than *US11* [47]. In contrast to *US2* and *US11*, *US3* retains MHC class I heterodimers loaded with peptides in the endoplasmic reticulum [41], whereas *US6* prevents the peptide transport through TAP [48]. By down-regulation of MHC class I molecules on the cell surface by expression of *US2*, *US3*, *US6*, and *US11*, infected cells evade CD8+ cytotoxic T cell recognition. CD8+ cytotoxic T cells play a significant role in immune recognition and defense mechanisms against most viruses, as infected cells can be lysed after presenting virus-derived antigen peptides with the help of MHC class I molecules on the cell surface [49]. A decrease in numbers of specific CD8+ lymphocytes in mice was observed after infection with HCMV virus vaccines that expressed *US2* and *US11*. The reduction of CD8+ lymphocytes as indicator for a specific cellular immune response ranged between 25% and 50% [47]. Further studies pointed out that cells transfected with HCMV *US2*, *US3*, *US6*, and *US11* showed reduction of MHC class I on the cell surface of human neuronal stem cells ranging from 20% to 50% compared to non-transfected cells as control [50]. One major gain of sufficiently reduced levels of MHC class I surface expression and US gene expression is the fact that modified cells can still be recognized and eliminated by natural killer cells (NK) [51,52,53,54].

The objective of this study was to combine the gains of one clinically well-established matrix, Matriderm^®^, with the prospects of genetically modified keratinocytes as a potential approach for an immune compatible full-thickness skin substitute with biological functionality. Viral vectors, containing the *US11* gene of HCMV, were generated and tested to determine their functionality. For sufficient immune escape, *US11* should down-regulate MHC class I surface expression in transfected keratinocytes, but at the same preserve a substantial amount of ligands as inhibitors for NK cell activation. Prior to transfection, human leukocyte antigen typing of primary keratinocytes was performed to ensure that the antibody bonding was specific. The effect of *US11* transfection was monitored with Western blotting and specific immunofluorescence staining and transient MHC class I surface reduction was observed at different points in time over a period of 48 h. In further steps, data was completed with flow cytometry to prove and quantify the down-regulation of MHC class I. This was followed by seeding of Matriderm^®^ sheets with transfected keratinocytes as well as HaCaT cells as well characterized human keratinocyte cell line [55]. The HaCaT cell line was included in experiments as positive control for successful seeding of Matriderm^®^ constructs. Cell viability and histological assessments of seeded Matriderm^®^ constructs showed long-term cell survival and histological as well as morphological patterns comparable to healthy human skin. The data presented in this in vitro study will; therefore, lay the foundations for future in vivo studies to determine the potential for clinical applications.

## 2. Results

**Figure 1:**
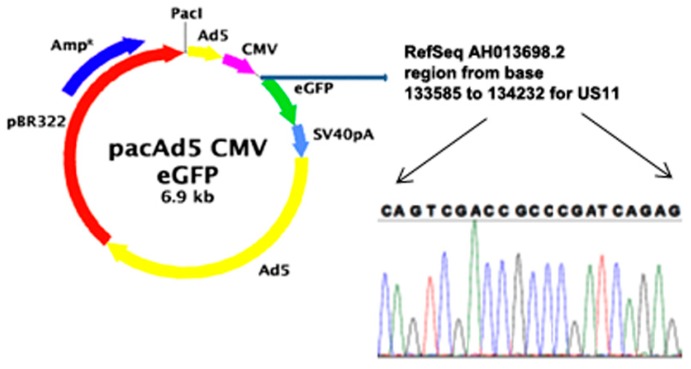
Sequence of viral pCMV6-US11-vectors. Displays the sequence of viral pCMV6-US11-vectors used in the study at hand. *US11 vector* was cloned into CMV between CMV and eGFP sequences (blue arrow). After cloning, whole genome sequencing was performed on the vectors. A short sequence is displayed between the black arrows (blue: cytosine, green: adenine, black: guanine, red: thymine).

In the present study, primary keratinocytes were successfully transfected with viral pCMV6-US11-vectors (Figure 1), which had been previously synthesized and were commercially available. All procedures followed standardized operating protocols according to the manufacturer’s instructions and internal protocols. Prior to transfection, the human leukocyte antigen typing of primary keratinocytes was investigated to ensure that antibody bonding was specific for MHC class I, HLA-A, and HLA-B on the cell surface. HLA-typing was performed by the Institute of Transfusion Medicine at Hannover Medical School and showed that primary keratinocytes expressed subtypes of HLA-A, HLA-B, HLA-C, and HLA-DRB1 on the cell surface (Table 1). Therefore, specific bonding of antibodies can be assumed for all of the following experiments.

### 2.1. Transfection with US11 Vectors Induced Transient MHC Class I Down-Regulation

**Figure 2:**
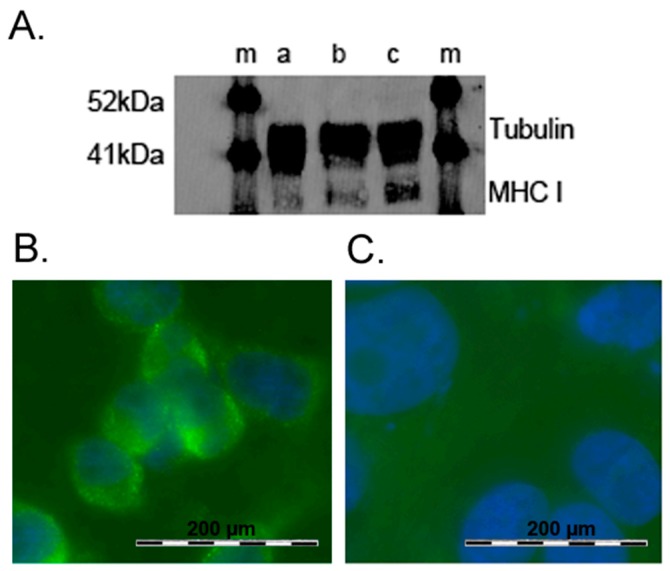
Western Blot and Immunofluorescence analysis. (**A**) Total protein extracts of primary keratinocytes were subjected to Western blotting following immunological detection of MHC class I to examine the overall expression levels post-transfection. Tubulin served as control marker (**m**). MHC class I expression levels were examined after 24 h (**a**) and 48 h (**b**) post-transfection, respectively, and were compared to non-transfected control (**c**). (**B**) In the control group, non-transfected primary keratinocytes were stained with indirect immunofluorescence staining. MHC class I expression is indicated by green fluorescent light emission in fluorescence microscopy. Samples were counterstained with (4′,6-Diamidino-2-Phenylindole (DAPI), indicated by blue fluorescent light emission of cell nuclei. (**C**) Transfected primary keratinocytes were stained with indirect immunofluorescence staining after 24 h post-transfection and showed reduced expression levels of MHC class I on the cell surface. The MHC class I expression is indicated by green fluorescent light emission in fluorescence microscopy. The samples were counterstained with DAPI, as indicated by blue fluorescent light emission of cell nuclei.

As seen in Figure 1, the enhanced green fluorescent protein (eGFP) was incorporated into the vectors used in the present study. Therefore, successful transfection could be detected by green fluorescent light emission (488 nm) of primary keratinocytes in fluorescence microscopy 24 or 48 h post-transfection (images not shown). The transfection efficiency was determined to at least 85% for all of the following experiments. To evaluate the consequences of transfection and to demonstrate the successful down-regulation of MHC class I molecules, indirect immunofluorescence staining was performed on transfected primary keratinocytes after 24 h and compared to non-transfected cells serving as control group. MHC class I specific antibodies were used for immunofluorescence staining as outlined above. First of all, cells of the control group (i.e., non-transfected primary keratinocytes) were stained with indirect immunofluorescence and showed bright MHC class I expression on the cell surface, indicated by green fluorescent light emission (Figure 2B). In comparison, transfected primary keratinocytes were stained with immunofluorescence staining using identical protocols after 24 h post-transfection. Modulation of MHC class I molecules expression was observed after 24 h and showed reduced MHC class I expression on the cell surface of a fraction of cells, indicated by decreased green fluorescent light emission in fluorescence microscopy (Figure 2C). In addition to immunofluorescence staining, Western blotting was performed on primary keratinocytes after 24 and 48 h post-transfection to further analyze the results of *US11* transfection. After protein extraction of primary keratinocytes and electrophoresis, immunoblotting was performed with a monoclonal rabbit Anti-MHC class I + HLA-A + HLA-B antibody as mentioned above. Tubulin served as a ubiquitous occurring control marker. The intensity of pictured protein bands is proportional to protein concentrations in samples and Western blotting can; therefore, be used for semi-quantitative predictions for MHC class I expression levels. As seen in Figure 2A, MHC class I proteins were reduced after 24 h (Figure 2A, a) as well as 48 h (Figure 2A, b) post-transfection with *US11* vectors compared to non-transfected control (Figure 2A, c). It is of interest that MHC class I protein quantity increased after 48 h compared to 24 h post-transfection. This is indicated through an increased protein band as seen in Figure 2A, b. Therefore, transient MHC class I down-regulation in a fraction of primary human keratinocytes can be assumed after transfection with *US11* vectors.

**Figure 3:**
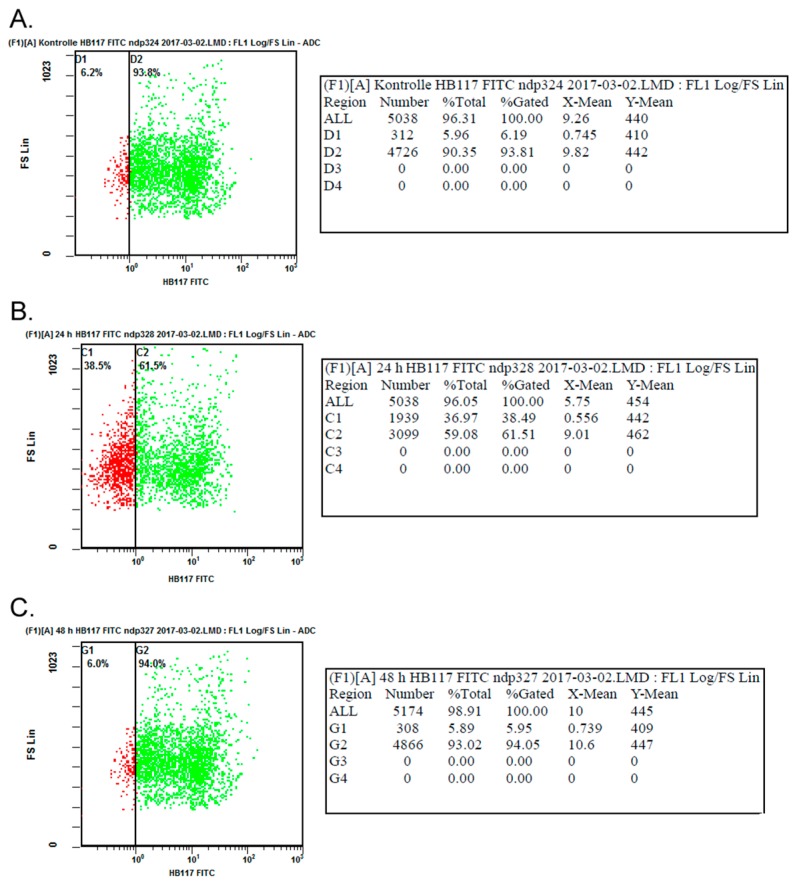
Flow cytometry. Each measuring point represents one measured cell. Cells with MHC class I expression on the cell surface are marked in green, whereas cells with no expression of MHC class I on the cell surface are marked in red. Flow cytometric analysis was performed on non-transfected primary keratinocytes as control after 24 h (**A**) as well as 48 h (data not shown). Further flow cytometric analysis was performed on primary keratinocytes 24 h (**B**) and 48 h (**C**) post-transfection and results were compared. (FITC: Fluorescein isothiocyanate; LMD: file format provided by Beckman Coulter (Krefeld, Germany); FS: forward scatter).

For further quantification and to prove MHC class I down-regulation initiated by *US11*, flow cytometry was performed on transfected primary keratinocytes after 24 and 48 h respectively. A total of 5000 transfected cells were selected for flow cytometric analysis and all procedures followed standardized operating protocols and good manufacturing practice. Primary antibodies were identical to those used for immunofluorescence staining and Western blotting. Non-transfected primary keratinocytes served as control. At the outset, non-transfected primary keratinocytes were analyzed after 24 and 48 h. As seen in Figure 3A, 93.8% of measured cells showed MHC class I expression on the cell surface after 24 h, whereas 6.2% of measured cells showed no MHC class I expression at all. Another measurement of non-transfected keratinocytes after 48 h showed comparable results (i.e., 94.0% of measured cells showed MHC class I expression on the cell surface; data not shown). Compared to controls, transfected primary keratinocytes were measured after 24 h post-transfection, respectively. As expected, of all measured cells 61.5% showed MHC class I expression on the cell surface, whereas 38.5% showed no MHC class I expression (Figure 3B). Therefore, a transient down-regulation in a fraction of transfected keratinocytes can be observed after 24 h post-transfection and the relative reduction of MHC class I expression can be calculated to 32.3%. Further flow cytometric analysis of transfected keratinocytes after 48 h showed an expression of 94.0% of MHC class I on the cell surface of all measured cells (Figure 3C). Therefore, an increase of expression of MHC class I can be postulated after 48 h compared to the expression levels after 24 h post-transfection. Furthermore, the MHC class I expression recovers to expression levels comparable to control measurements of non-transfected keratinocytes after 48 h post-transfection.

### 2.2. Seeding of Matriderm^®^ Constructs and Evaluation of Long-Term Cell Survival

**Figure 4:**
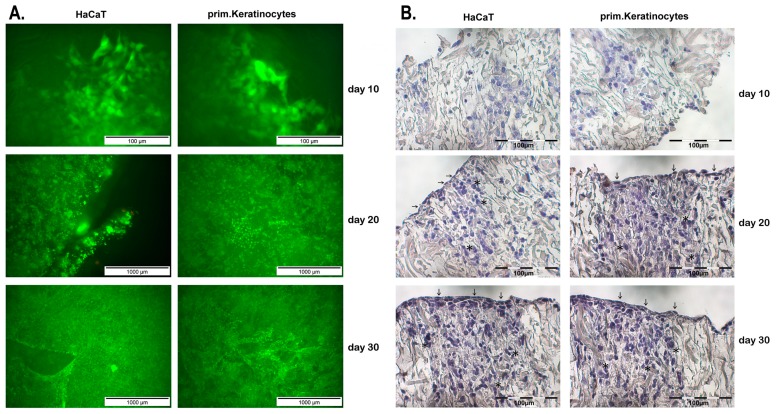
Live–dead assay and histological analysis. (**A**) Transfected primary keratinocytes as well as HaCaT cell line were seeded on Matriderm^®^ sheets and cultured. Cell survival was observed after 10, 20, and 30 days using live–dead assays. Viable cells were visualized by green fluorescent light emission compared to dead cells visualized by red fluorescent light emission. (**B**) Haematoxyline and eosine staining was performed on sections of primary keratinocyte or HaCaT cell line-seeded Matriderm^®^ constructs. Histological staining was performed after 10, 20, and 30 days of culture and results were compared. On the matrix surface, cells showed a more flattened and elongated cell morphology (arrows), whereas inside of the Matriderm^®^ constructs cells showed a more roundish cell morphology (stars).

Transfected primary keratinocytes as well as HaCaT cell line were seeded on Matriderm^®^ sheets with a thickness of 1 mm and cultured over a period of 30 days. To evaluate cell survival over this period of time, life-dead-assays were performed after 10, 20, and 30 days. Viable cells were stained with calcein-acetomethylester to indicate intracellular esterase activity and could be visualized by green fluorescent light emission (488 nm) by fluorescence microscopy. Dead cells were stained with ehtidium-homodimer-III to indicate the loss of plasma membrane integrity and were visualized by red fluorescent light emission (546 nm). Viable cells could be observed in HaCaT-seeded Matriderm^®^ constructs after 10 days. Only a negligible number of cells were dead. Even after 20 and 30 days almost all cells continued to show as viable (Figure 4A). Viable HaCaT cells were distributed throughout the whole matrix after 10, 20, and 30 days. The density of cells increased over the period of 30 days, which led us to postulate that transfected HaCaT cells devised and proliferated (Figure 4A). Furthermore, viable primary keratinocytes could be observed in seeded Matriderm^®^ constructs after 10, 20, and 30 days with only a negligible number of dead cells. Viable primary keratinocytes were observed throughout the whole matrix and whilst an increase in cell density could furthermore be examined (Figure 4A). To sum up, no differences between HaCaT or primary keratinocytes seeded Matriderm^®^ sheets were observed over a period of 30 days in terms of cell viability, cell distribution in the matrix as well as cell proliferation. Cells migrated throughout the whole matrix in longitudinal and transversal directions.

### 2.3. Histological Patterns and Cell Morphology after Seeding of Matriderm^®^ Constructs

Haematoxyline and eosine staining was performed on sections of Matriderm^®^ constructs after 10, 20, and 30 days of culture to evaluate cell morphology, cell distribution, and matrix composition over different time periods. In haematoxyline and eosine staining (H&E), nuclei are stained bluish-violet, cytoplasm and collagen fibers red, whereas elastic fibers are either unstained or light rose. After 10 days, isolated roundish HaCaT cells could be observed in Matriderm^®^ constructs without formation of united cell structures and distributed throughout the whole matrix as could be demonstrated with live–dead assays (Figure 4B, stars). At the same time, isolated reddish staining of the matrix was observed after 10 days (Figure 4B, stars). After a period of 20 days, cell density of HaCaT increased and cells started forming united cell structures. Cell morphology of HaCaT cells on Matriderm^®^ surface differed from cell morphology inside the matrix. On the matrix surface, a more flattened and elongated cell morphology could be observed (Figure 4B, arrows), whereas inside of the Matriderm^®^ constructs cells showed a more roundish cell morpholohy (Figure 4B, stars). After 30 days, cell density of HaCaT further increased and distinctive united cell structures continued to be visible inside and on the surface of Matriderm^®^ constructs. On Matriderm^®^ surface, continuous bluish united flattened cell structures were observed that increased in thickness compared to constructs after 20 days (Figure 4B, arrows). The reddish staining of the matrix increased in parallel with increasing cell density and formation of united cell structures after 20 and 30 days (Figure 4B). The number of HaCaT cells increased from basal to apical in Matriderm^®^ constructs over the course of 30 days.

In addition, Matriderm^®^ constructs were also seeded with transfected primary keratinocytes and histological sections were stained with HE after 10, 20, and 30 days. Histological analysis revealed identical results compared to HaCaT seeded Matriderm^®^ constructs. In short, after 10 days isolated roundish primary keratinocytes could be observed throughout the whole matrix (Figure 4B, stars). Isolated reddish staining of the matrix was observed as well. After 20 days, cell density increased, cells formed first united cell structures and, from basal to apical, primary keratinocytes changed from roundish cell morphology (Figure 4b, stars) to a more flattened and elongated appearance (Figure 4B, arrows). On the matrix surface, united flattened cell structures were observed for the first time (Figure 4B, arrows). After 30 days, cell density of primary keratinocytes further increased and went hand in hand with a rising number of united cell structures, as well as an increasing reddish staining of the matrix. The number of primary keratinocytes increased from basal to apical in Matriderm^®^ constructs and on the matrix surface the united flattened cell structure increased in thickness (Figure 4B, arrows).

## 3. Discussion

To author’s knowledge, this is the first study that describes the successful targeted gene modification of primary keratinocytes with transfection of virus-encoded *US11* of HCMV. Herpes viruses, such as HCMV, are remarkably successful in expressing immune evasive strategies utilizing genes, such as *US11*, that encode proteins that interfere with MHC class I mediated antigen presentation. In the study at hand, keratinocytes were transfected with *US11* to inhibit the MHC class I antigen processing pathway as a potential approach for reducing the immunogenicity of gene-modified cells [56]. Transfected keratinocytes were first generated, and further investigations showed transient down-regulation of MHC class I in a fraction of transfected primary keratinocytes. Initially, HLA-typing of primary keratinocytes was performed and showed subtypes of HLA-A, HLA-B, HLA-C, and HLA-DRB1 on the cell surface. It is noteworthy that the success of immune escape of HCMV through modulation of MHC class I surface expression is likely to be influenced by MHC class I allele specificity of different US proteins. In general, it is believed that HLA-A and HLA-B alleles are down-regulated, but evidence is scarce [57,58,59]. Further studies showed that all HLA-A, most -B, all -G, and no -C or -E alleles are down regulated by *US2* [57]. Therefore, flow cytometry analyses seem to be a valuable complement to experiments to prove down-regulation of MHC class I by *US11* transfection. Nevertheless, precise data is scarce and more experiments are needed.

As the results of immunofluorescence staining as well as Western blotting showed, a fraction of primary keratinocytes transfected with virus-encoded *US11* of HCMV had decreased expression levels of MHC class I after 24 and 48 h. Flow cytometry further quantified the decrease of MHC class I surface expression from 93.8% to 61.5% after 24 h post-transfection of all measured cells, indicating a relative decline of 32.3%. Therefore, a decrease of MHC class I expression could be observed in a fraction of transfected primary keratinocytes. The successful decrease in MHC class I surface expression after 24 h can be traced back to the effect of *US11* that redirects newly synthesized MHC class I heavy chains from the endoplasmic reticulum to the cytosol, where they are degraded by the proteasomes as described elsewhere [43,44,45]. The aim for sufficient immune escape by transfecting primary keratinocytes with *US11* gene of HCMV is; therefore, reached after 24 h in a fraction of cells. A comparison of this study’s results to other findings is difficult, as to date there are no other studies that quantify the expression levels of MHC class I in primary keratinocytes after viral transfection with *US11*. It was earlier reported that MHC expression in a human adult stem cell line could be down-regulated by approximately 40% compared to control groups by transfection with the *US11* gene [60]. Human neuronal stem cells transfected with *US2*, *US3*, *US6*, and *US11* showed decreased MHC class I expression in flow cytometry, ranging from 20% to 50% compared to controls [50]. Other studies demonstrated significant variability in reducing MHC class I expression on the cell surface of different human cell lines by transfection with the *US11* gene of HCMV. To sum up, all human cell lines transduced with the *US11* gene showed reduced expression levels of MHC class I, ranging from 64% decrease in VA13 fibroblasts to 96% decrease in MOLT-3 cells [61]. Therefore, the cell type specific variability of reduction of MHC class I after *US11* gene transfection may limit immune evasive properties in some cell types. Regarding the point in time of transcript expression after transfection with *US11*, the results presented correspond to the results reported elsewhere. It is believed that *US2* and *US11* transcripts appear approximately 6 h after infection [56]. The study at hand showed reduced MHC class I expression levels in a fraction of transfected cells after 24 h, without further quantifying expression levels below the period of 24 h.

It is noteworthy that in the study at hand flow cytometric and Western blotting results of transfected keratinocytes presented increasing expression levels of MHC class I after 48 h post-transfection. A total of 94.0% of all measured cells showed MHC class I expression 48 h post-transfection and thereby identical MHC class I expression compared to non-transfected control cells. The results presented stand in contrast to other studies that determined a consistent decrease in MHC class I expression on the cell surface in A375 human malignant melanoma cell line over a period of six months [61]. Based on the results presented, one can postulate that *US11* expression in transfected primary keratinocytes is only transient in a fraction of cells for at least 24 h but less than 48 h. One possible explanation could be cutbacks of viral vectors in primary keratinocytes with re-increasing levels of MHC class I on the cell surface of primary keratinocytes. On the other hand, Radosevich et al. pointed out that different human cell lines showed a significant variability in reducing MHC class I expression on the cell surface by transfection with *US11* [61]. Therefore, primary keratinocytes might not be an ideal cell source for immune evasion with *US11*. As reported elsewhere, host cytokine release after transgene exposure with *US11* was observed and thus might lead to an up-regulation of MHC class I expression [61]. This may complicate the use of potential applications of *US11* transfection for immunological modification of keratinocytes for further in vivo experiments. As a consequence, further studies are needed to quantify the effects of *US11* transfection on MHC class I expression levels in human primary keratinocytes, focusing especially on long-term effects of viral vector expression. Real-time polymerase chain reaction could be a valuable complement to experiments to access the effects of *US11* on biomolecular levels.

Another possible explanation for the transient nature of MHC class I down-regulation in primary keratinocytes might be related to the viral vector used in the study at hand. It was earlier observed elsewhere that viral vectors failed to persist in host cells and that transgene expression was typically short-lived. Over the past few years, substantial progress has been made and gene transfer technologies have been improved to allow a more efficient, specific, and safe application of viral vectors [62]. Vector tropism, the duration of transgene expression, as well as vector immunogenicity are crucial issues for the suitability of a vector for specific transgene applications. Adenovirus vectors showed a broad tropism and turned out to be extremely efficient in the transduction of different tissues and cells [63,64,65]. At the same time, the high inflammatory potential of adenovirus vectors has to be considered [66,67]. Lentivirus vectors could be another potential approach towards an efficient transfection protocol of primary keratinocytes because a persistent gene transfer could be observed in the absence of inflammation [68,69]. However, lentivirus vectors share the disadvantage of induced oncogenesis in some applications [70]. Herpesvirus vectors, such as the *US11* vector used in the present study, share the advantages of large packaging capacity and an moderate inflammatory response [71,72,73]. Given the diversity of viral vectors available, herpesvirus derived vectors represent the most auspicious approach towards an efficient and specific transfection protocol of primary keratinocytes. In addition, cell clones who expressed a stable *US11* expression post-transfection could be isolated in the present study. The clones are currently part of further research to establish a transfection protocol with stable transcript expression.

A remaining question is to what level MHC class I has to be decreased in vivo to evade host CD8+ cytotoxic T cell recognition or NK cell activation. It was reported that as little as one to 200 MHC class I molecules presented per cell are sufficient to mount a host response [74,75], whereas complete or near-complete elimination of MHC class I presentation on the cell surface is known as catalyst for NK cell mediated apoptosis [76]. Previous reports indicated that there is a correlation between MHC class I expression on the cell surface and the susceptibility to NK cell induced apoptosis [77]. Taken these findings together, further experiments are needed to evaluate cytotoxic effects of *US11* transfected keratinocytes. Furthermore, immune evasive strategies have to be improved to prevent both NK cell and CD8+ cytotoxic T cell recognition using viral vectors such as *US11*. For future studies, it could be feasible to combine the co-expression of *US3* and *US11* to induce efficient robust MHC class I down-regulation as described elsewhere [60,78]. Furthermore, future approaches have to be conducted with regard to up-regulating mechanisms that enhance MHC class I presentation on the cell surface such as multiple cytokines [79]. Another potential approach suggested by recent studies is combining immune evasive strategies of human immunodeficiency virus (HIV) Nef protein in combination with HCMV *US2* and *US11* [80,81]. However, this approach has a multitude of potential disadvantages, such as increased immunogenicity when utilizing two different immune evasion genes. Thus, it is more useful to use a single viral vector to reach sufficiently decreased MHC class I expression levels on the cell surface if improved immune evasive capacities could be achieved [82]. Gene therapy for the skin using gene editing systems such as Clustered Regularly Interspaced Short Palindromic Repeats (CRISPR/Cas9) is also an up-coming, promising alternative for gene modification, which however neglects the risks associated with viral vectors [83,84,85].

Further experiments in the present study focused on seeding of Matriderm^®^ sheets with transfected keratinocytes in order to evaluate the potential use as on-the-shelf transplantable skin substitute. A HaCaT cell line was included in experiments as positive control for the successful seeding of Matriderm^®^ constructs. It is of interest that other work groups described difficulties with the seeding of Matriderm^®^ with keratinocytes [15] and pointed out that cell migration to internal matrix parts is a crucial issue for manufacturing tissue-engineered constructs with high biological functionality [18]. However, as mentioned above, the results of the study at hand showed viable transfected keratinocytes as well as HaCaT cell line in seeded Matriderm^®^ constructs over a period of 30 days, with no significant differences in experimental groups versus control groups. Cells were distributed throughout the whole matrix and no difficulties in seeding of Matriderm^®^ constructs were observed. Furthermore, cell proliferation and migration, indicated by an increasing number of cells, could be observed in all experimental groups as well as control groups. As cell migration is crucial for the biological functionality of the substitute, one can conclude the Matriderm^®^ construct presented in this study bears great potential for overcoming the problems described elsewhere [15,18]. The observed results in terms of cell viability correspond to the results of other work groups who even used different thicknesses of Matriderm^®^ sheets for seeding with keratinocytes [15].

Histological results proved live–dead assays in terms of increased cell density and showed cell migration throughout the whole matrix in both experimental and control groups with no significant differences. Cells formed united cell structures and as they transitioned from basal to apical, the cells changed their morphology from a roundish to more flattened appearance. Furthermore, after 30 days of culture on the matrix surface, the united flattened cell structures increased in thickness and formed a newly synthesized keratinocyte layer on the matrix surface. In comparison to healthy human skin, there are some similarities to our primary keratinocyte seeded Matriderm^®^ construct. In healthy human skin, epidermal layers consist mainly of keratinocytes, whereas dermal layers include vascular components and fibroblasts that synthesize the extra cellular matrix [86]. Keratinocytes play a significant role in restoring the epidermal barrier to prevent further fluid loss or microbial infections [87], but wound closure after full-thickness skin injuries requires, at a minimum, to reestablish a stable epidermis [11]. The Matriderm^®^ construct developed in the present study consists only of keratinocytes, but a complete restoration of epidermal layer could be histologically observed. However, epithelial closure does not restore the anatomy and physiology of the skin, because for full restoration all cell types of the skin have to be present in tissue engineered constructs [88]. For example, there is still a lack of cutaneous structures or functions, such as dermal-epidermal junction, hair follicle genesis, subcutaneous and sweat glands, pigmentation, as well as sensorimotor innervation [11].

Furthermore, functional and aesthetical results have to be considered for future applications of the construct as it is presented here, as wound contraction is another crucial issue of skin tissue engineering. However, wound healing is a complex process that covers multi-level, temporally overlapping mechanisms such as inflammation, blood clotting, cellular proliferation, and extracellular matrix remodeling [89,90]. Especially regarding the skin, wound healing differs between species. For example, lower vertebrates such as *axolotls* possess the ability to perfectly regenerate their entire skin, including secretory appendages [91]. In rodent models that are commonly used as first step for in vivo observations, wound contraction is one major mechanism of wound closure [92,93], whereas in humans re-epithelialization and formation of granulation tissue are the major mechanisms [89,90]. Another work group developed a rodent model that closely parallels human wound healing by showing that splinted wounds in rodents have an increased amount of granulations tissue without affecting the rate of re-epithelialization compared to control groups [93]. Although the present study observed no contraction of Matriderm^®^ constructs, further in vivo studies are needed to evaluate contraction properties. As other studies pointed out, the mouse dorsal skin fold chamber for tissue engineered skin could be an appropriate tool for in vivo testing. The model is well-suited because dorsal skin fold chambers reduce wound contraction to a minimum and a sophisticated analysis of constructs in vivo is feasible [19,94].

Another issue in skin tissue engineering is neovascularization and lymphangiogenesis. Both mechanisms are essential for survival of skin grafts and dermal substitutes from the biological and surgical perspective [95,96,97,98]. Autologous skin grafts are vascularized through angiogenesis and vasculogenesis starting at the periphery of the wound after three days and moving towards the center of the wound after 21 days, replacing donor graft vasculature through recipients endothelial cells [99]. In contrast to skin grafts, the vascularization of dermal skin substitutes is prolonged and shows slow vascularization kinetics [100]. Due to high biocompatibility and availability, Matriderm^®^ grafts are commonly used as templates for dermal reconstruction with immediate split-thickness skin grafting coverage; therefore, representing an ideal matrix for skin tissue engineering applications [9,12,13,14]. It was recently reported that cell-free collagen type I-coated Matriderm^®^ constructs transplanted to dorsal skin fold chambers in mice showed promising results in terms of integration into the surrounding tissue, wound contractions, and neovascularization. However, the observation period of 11 days was too short to observe full epithelial regeneration [19]. The results of the study at hand, such as the demonstrated long-term survival of cells over a period of 30 days and the histological patterns comparable to epidermal layers of healthy human skin, constitute promising evidence that could provide the basis for further work towards vascularization and angiogenesis in vivo.

**Figure 5:**
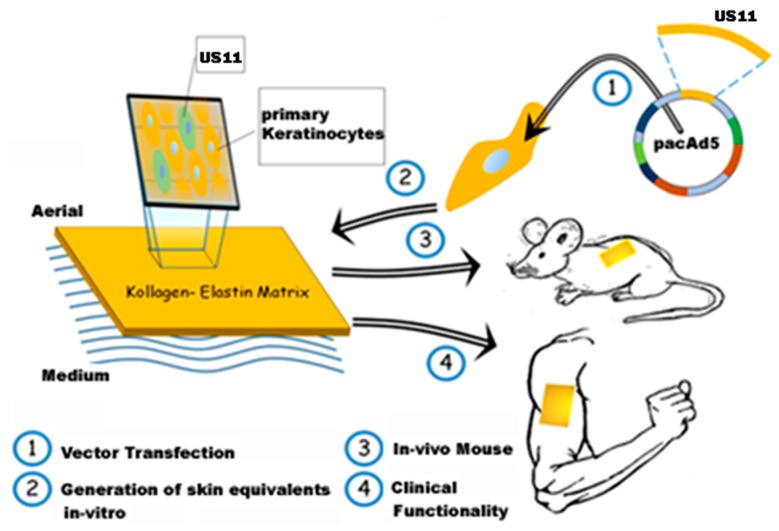
Schematic outline for future experiments. Displays the outline for further in vivo experiments as well as potential future clinical applications. Nevertheless, further data is needed to evaluate the potential of the results presented in the study at hand.

The data presented in the study at hand lay the foundation for continuative research to further quantify the effect of *US11* transfection in primary keratinocytes, and to establish a transfection protocol that guarantees a more stable expression of transcripts after transfection. The promising results of this study show that, in the long-term, an immune-privileged novel skin substitute based on Matriderm^®^ matrices and transfected primary keratinocytes might be available. Potential future clinical applications are allo-transplantations of human skin or keratinocytes to treat severe burn injuries in immunocompromised burn patients disregarding the possibility of graft rejection (Figure 5). However, additional data, especially for long-term observations in vivo, is needed to determine the immunological compatibility as well as sufficient vascularization and sufficient re-epithelialization for Matriderm^®^ constructs developed in the study at hand. The data presented here will support future experiments to evaluate the in vivo potential of the developed skin substitute in a rodent model. In addition, further assessments are needed to evaluate the potential use for future clinical applications (Figure 5).

## 4. Materials and Methods

### 4.1. Cell Lines and Cell Culture

The human primary dermal keratinocytes (Provitro, Berlin, Germany) and HaCaT cell line (Cell Line Services, Eppelheim, Germany) used in this study were commercially available. According to the data sheet, the primary keratinocytes had been isolated from human skin tissue donated by a Caucasian female aged 40 years. The HaCaT cell line had been isolated from human skin tissue donated by a Caucasian male aged 62 years according to Boukamp et al. [55]. The study was conducted in accordance with the Declaration of Helsinki and the protocol was approved by the Ethics Committee of Hannover Medical School (Approval Number: 2520-2014, Approval Date: 5 January 2017). Keratinocytes were maintained in serum-free Keratinocyte growth medium (Provitro, Berlin, Germany), which was supplemented with manufacturer-provided supplements according to manufacturer’s instructions. HaCaT were cultured in Dulbecco’s Modified Eagle Medium (DMEM) medium supplemented with 4.5 g/L glucose, 2 mM L-glutamine, and 10% fetal bovine serum (Cell Line Services, Eppelheim, Germany) according to manufacturer’s instructions. Cells were incubated at 37 °C and 5% CO_2_ in a humidified atmosphere. Upon reaching confluence, cells were detached with Dispase II (Provitro, Berlin, Germany) and sub-cultured.

### 4.2. Human Leukocyte Antigen Typing of Primary Keratinocytes

Human leukocyte antigen typing (HLA-typing) of primary keratinocytes was performed by the Institute of Transfusion Medicine at Hannover Medical School (Hannover, Germany). Procedures followed standard operating protocols and good manufacturing practice.

### 4.3. Vector Transfection

Primary keratinocytes were transfected with pCMV6-US11-vectors (ORIGENE, Rockville, MD, USA) and the appropriate control vectors by the use of Turbo Fectin 8.0 Transfection Reagent (AMS Biotechnology, Ambingdon, UK) according to the manufacturer’s instructions. The cells were seeded with a cell density of 2 × 10^4^ cells/cm^2^ and incubated at 37 °C in a humidified atmosphere with 5% CO_2_ for 24 and 48 h before being analyzed. To determine the transfection efficiency, transfected primary keratinocytes underwent inverse fluorescence microscopy with a Zeiss Axiovert 200 M microscope (Carl Zeiss, Oberkochen, Germany) 24 or 48 h post-transfection. Successful transfection could be detected by green fluorescent light emission caused by enhanced green fluorescent protein (eGFP) that was incorporated into the pCMV6-US11-vectors. The images are not shown in the manuscript. A transfection efficiency of at least 85% was assumed for all further experiments. Non-transfected cells served as control.

### 4.4. Immunofluorescence

To prove successful down-regulation of MHC-I-receptors, indirect immunofluorescence staining was performed on transfected primary keratinocytes after 24 h as well as non-transfected cells as control. Cells were fixed with 4% paraformaldehyde (Sigma Aldrich, Darmstadt, Germany) in PBS (Sigma Aldrich, Darmstadt, Germany), permeated with 0.2% Tritron X-100 (Carl Roth, Karlsruhe, Germany), and blocked with 2% fetal calf serum (Biochrom, Berlin, Germany). The primary antibody used in this study was monoclonal rabbit Anti-MHC class I + HLA-A + HLA-B antibody (abcam, Cambridge, UK). The primary antibody was applied for 1 h, followed by extensive washing with phosphate buffered saline (PBS). Alexa Fluor^®^ 488 conjugated anti-rabbit antibody (Invitrogen, Carlsbad, CA, USA) was used as secondary antibody. Samples were counterstained with DAPI. Inverse fluorescence microscopy was performed with a Zeiss Axiovert 200 M microscope (Carl Zeiss, Oberkochen, Germany) and associated software.

### 4.5. Western Blotting

Total protein extract of primary keratinocytes was made in a radioimmunoprecipitation assay buffer (RIPA) containing 0.3 M NaCl, 1% sodium desoxycholate, 0.1% sodium dodecyl sulfate (SDS), 1% Triton-X-100, 20 mM Tris–HCl (pH 8), and 1 mM ethylene diamine tetraacetic acid (EDTA) supplemented with 1 mM phenylmethyl sulfonyl fluoride (PMSF). A total of 25 µg of each protein sample were separated by electrophoresis on 15% SDS–polyacrylamide gels and then transferred on a polyvinylidene fluoride (PVDF) membrane (Millipore Corporation, Bedford, MA, USA). Immunoblotting was performed with monoclonal rabbit Anti-MHC class I + HLA-A + HLA-B antibody (abcam, Cambridge, UK). Odyssey 680/800 nm secondary conjugates (Li-Cor BioSciences, Lincoln, NE, USA) were used for the quantification of protein expression levels and signals were visualized using the Odyssey Infra-Red Imaging System and software (Li-Cor BioSciences, Lincoln, NE, USA).

### 4.6. Flow Cytometry

Transfected primary keratinocytes (passage 2) were examined for MHC I markers using flow cytometry. Non-transfected keratinocytes served as control. According to standardized protocols, 5000 successfully transfected primary keratinocytes were selected for flow cytometric analysis. For immunolabelling, cells were detached from culture flasks with Dispase II (Provitro, Berlin, Germany) and washed with phosphate-buffered saline (Sigma Aldrich, Darmstadt, Germany) containing 10 % FBS. Then, cells were blocked with 1% BSA (Sigma Aldrich, Darmstadt, Germany) in PBS for 30 min at 4 °C, centrifuged for 5 min at 300× *g*, resuspended in PBS, and incubated with the primary antibodies (monoclonal rabbit Anti-MHC class I + HLA-A + HLA-B antibody (abcam, Cambridge, UK), final dilution 1:10, for 50 min at 4 °C. After washing with PBS, cells were incubated with fluorochrome labeled secondary antibodies (Santa Cruz, Heidelberg, Germany), final dilution 1:5, for 45 min at 4 °C, washed and then analyzed. For measurement, a FC500 flow cytometer (Beckman Coulter, Krefeld, Germany) was used.

### 4.7. Matriderm^®^ Constructs

Matriderm^®^ (Medical Systems Solution, Oberentfelden, Switzerland) is a three-dimensional bovine-derived collagen-elastin matrix available in various thicknesses. For this study, 1 mm matrices were used. HaCaT cells and primary keratinocytes (passage 2) were seeded on Matriderm^®^ and incubated in serum-free Keratinocyte growth medium (Provitro, Berlin, Germany) at 37 °C and 5% CO_2_ in a humidified atmosphere for 10, 20, and 30 days before being analyzed with live–dead assay and histology.

### 4.8. Live–Dead Assay

Cell viability was evaluated by the use of LIVE/DEAD^®^ Viability/Cytotoxicity Kit for mammalian cells (Life Technologies, Darmstadt, Germany) according to the manufacturer instructions. The constructs were washed with PBS and incubated with staining solution (1 mL PBS with 1 μL calcein and 2 μL ethidium homodimere-III) for 30 min at room temperature. After two PBS washing steps, vital cells were capable of taking up calcein and could be analyzed by green fluorescent light emission (488 nm). Dead cells could be detected by red fluorescent signal (546 nm) under the fluorescent microscope (Olympus, Hamburg, Germany). The resulting images were overlaid.

### 4.9. Histology

After culturing time, Matriderm^®^ constructs were fixed with 4% buffered formalin (Carl Roth, Karlsruhe, Germany), dehydrated in a graded series of increasing alcohol concentration, cleared in xylene, embedded in standard procedure in paraffin, and cut into 10 µm sections with a microtome (Microm International GmbH, Walldorf, Germany). Slides were deparaffinated, rehydrated by descendent alcohol concentrations and stained with haematoxyline and eosine (H&E). For H&E staining, constructs were stained with 1% haematoxyline (Merck, Darmstadt, Germany) for 5 min, rinsed with tab water for 10 min, and stained with 2% eosine (Merck, Darmstadt, Germany) for an additional 2 min. Slides were dehydrated by ascendant alcohol concentration, mounted in Roti^®^-Histokit (Carl Roth, Karlsruhe, Germany), and analyzed with a Zeiss Axiovert 200 M microscope (Carl Zeiss, Oberkochen, Germany) and associated software.

## Figures and Tables

**Table 1:** Displays subtypes of human leukocyte antigens expressed by human primary keratinocytes. HLA-typing was performed prior to transfection to ensure that antibody bonding was specific for MHC class I, HLA-A, and HLA-B on the cell surface. HLA subtypes are named according to the HLA naming system developed by the World Health Organization Committee for Factors of the HLA System in 2010.

Human Leukocyte Antigen	Subtypes
HLA-A	*02, *11
HLA-B	*15:01:01G, *27
HLA-C	*02, *03
HLA-DRB1	*13, *16

## Abbreviations

MHC	Major Histocompatibility Complex
US	Unique Short Glycoprotein
TAP	Transporter-Associated-With-Antigen-Processing
β2-M	β2-Microglobulin
HLA	Human Leukocyte Antigen
HCMV	Human Cytomegalovirus
CD	Clusters of Differentiation
NK	Natural Killer Cell
eGFP	Enhanced Green Fluorescent Protein
DAPI	4′,6-Diamidino-2-Phenylindole
FITC	Fluorescein Isothiocyanate
LMD	File format provided by Beckman Coulter (Krefeld, Germany)
FS	Forward scatter
H&E	Haematoxyline and Eosine
CRISPR	Clustered Regularly Interspaced Short Palindromic Repeats
DMEM	Dulbecco’s Modified Eagle Medium
PBS	Phosphate Buffered Saline
RIPA	Radioimmunoprecipitation Assay Buffer
EDTA	Ethylene Diamine Tetraacetic Acid
PMSF	Phenylmethyl Sulfonyl Fluoride
PVDF	Polyvinylidene Fluoride
FBS	Fetal Bovine Serum
BSA	Bovine Serum Albumin
